# Spread of *E. coli* O157 infection among Scottish cattle farms: Stochastic models and model selection

**DOI:** 10.1016/j.epidem.2010.02.001

**Published:** 2010-03

**Authors:** Xu-Sheng Zhang, Margo E. Chase-Topping, Iain J. McKendrick, Nicholas J. Savill, Mark E.J. Woolhouse

**Affiliations:** a Centre for Infectious Diseases, University of Edinburgh, Kings Buildings, West Mains Road, Edinburgh EH9 3JT, UK; b Biomathematics and Statistics Scotland (BioSS), James Clerk Maxwell Building, Kings Buildings, Edinburgh, EH9 3JZ, UK

**Keywords:** *E. coli* O157, Transmission dynamics, Model fitting, Model selection, Risk factors

## Abstract

Identifying risk factors for the presence of *Escherichia coli* O157 infection on cattle farms is important for understanding the epidemiology of this zoonotic infection in its main reservoir and for informing the design of interventions to reduce the public health risk. Here, we use data from a large-scale field study carried out in Scotland to fit both “SIS”-type dynamical models and statistical risk factor models. By comparing the fit (assessed using maximum likelihood) of different dynamical models we are able to identify the most parsimonious model (using the AIC statistic) and compare it with the model suggested by risk factor analysis. Both approaches identify 2 key risk factors: the movement of cattle onto the farm and the number of cattle on the farm. There was no evidence for a role of other livestock species or seasonality, or of significant risk of local spread. However, the most parsimonious dynamical model does predict that farms can infect other farms through routes other than cattle movement, and that there is a nonlinear relationship between the force of infection and the number of infected farms. An important prediction from the most parsimonious model is that although only ∼ 20% farms may harbour *E. coli* O157 infection at any given time ∼ 80% may harbour infection at some point during the course of a year.

## Introduction

*Escherichia coli* O157 emerged over two decades ago and is now widespread in Scotland, where the incidence rate of human infection is generally higher than in most other UK, European or North American countries (Locking et al., 2006; Chase-Topping et al., 2008). Cattle are the main reservoir host for *E. coli* O157 (Armstrong et al., 1996) and play a significant role in the epidemiology of human infections (Griffin and Tauxe, 1991). Previous work has shown that direct contact with animals, their faeces and the farming environment are all important risk factors for sporadic human infections (O'Brien et al., 2001; Locking et al., 2001; Willshaw et al*.,* 2003). Spatial analyses also identified a positive association between human infections and areas of high cattle density (Michel et al*.,* 1999; Kistemann et al., 2004; Innocent et al., 2005). Although human infection may arise from person to person contact and from consumption of food contaminated by asymptomatic human carriers, primary human infection can be attributed to contamination of the environment or the food chain from several livestock species, especially cattle (Espie et al., 2006). Therefore understanding the mechanisms of persistence and spread of *E*. *coli* O157 on Scottish cattle farms is key to reducing the risk of human infection in Scotland and elsewhere.

Two recent surveys (Gunn et al., 2007; Pearce et al., 2009) concluded that c.20% of Scottish cattle farms harbour *E. coli* O157 infection, although there is variation in both time and space. Investigations with an exploratory mathematical model (Liu et al., 2007) suggested that although cattle movement is a significant contributor to the observed prevalence of *E. coli* O157 positive farms, it is not by itself sufficient to explain the persistence of *E. coli* O157 on Scottish cattle farms. The objective of this study is therefore to better understand the impact of other risk factors on the spread and persistence of *E. coli* O157 in Scotland and their interaction with cattle movement. To do this we developed a set of stochastic epidemiological models that represent different assumptions regarding the transmission of infection among farms. By comparing the goodness of fit of different models we gain insights into the underlying epidemiology of infection. Data were also analysed using traditional risk factor analysis in order to produce comparative results. Correspondence between the results from empirical statistical models and those from the fitting of dynamical process models has not, as far as we are aware, previously been examined in infectious diseases systems.

## Methods

*Data*. Three separate databases were used in this study. First, the June 2003 Agricultural census of livestock premises combined with the Department for Environment, Food and Rural Affairs (DEFRA) list of livestock premises; second, the Cattle Tracing System (CTS); and third, *E.coli* O157 prevalence data. Each data set will be described briefly below.

**Fig. 1:**
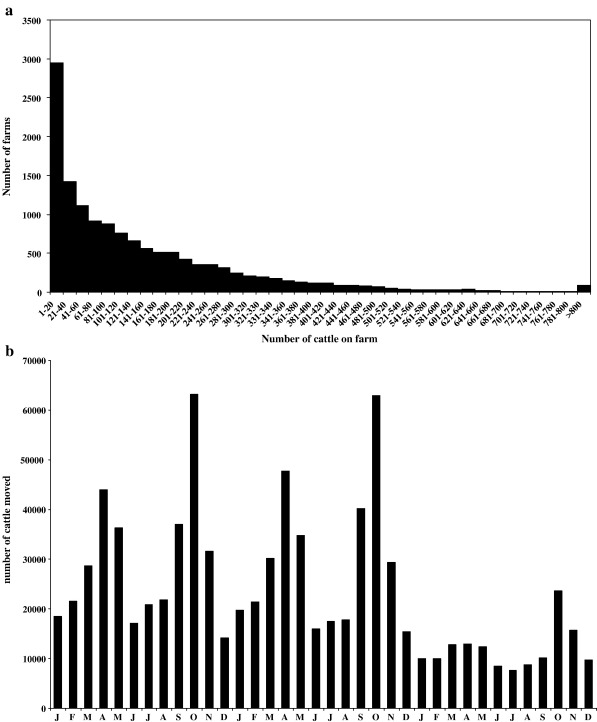

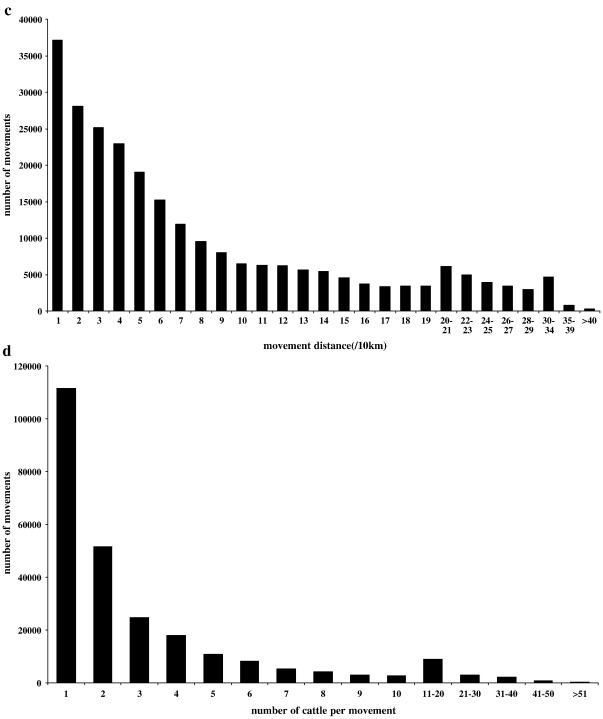
(a) distribution of farm sizes. (b) number of cattle moved between Scottish livestock farms each month from January 2002 to December 2004. (c) distances moved and (d) number of cattle moved per movement event from the same data set as (b).

**Fig. 2:**
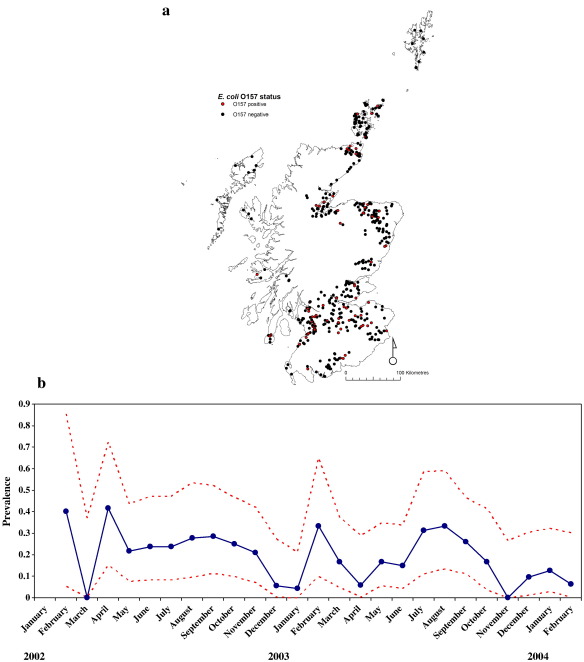
(a) geographical distribution of the 461 farms sampled in Scotland between February 2002 and February 2004. Red dots indicate positive farms. The prevalence is estimated as 18.9% (exact binomial 95% CI = 15.5 to 22.7). (b) temporal variation in prevalence of *E. coli* O157 infection. Black is the estimated prevalence for a given month and red dotted lines represent exact binomial 95% confidence intervals.

(1) June 2003 Agricultural census data (DEFRA, 2005). Among the original 50,266 farms in Scotland recorded in the census, 22,286 farms are provided with the numbers of animals but only 13,704 farms have cattle. Our system comprises these 13,704 cattle farms. The data include the Council-Parish-Holding number (CPH), the X–Y coordinates of the farm-house, the area of the farm, and the numbers of cattle, sheep and pigs. The farms are unevenly distributed across Scotland: with high densities in SW and NE Scotland and low densities in the Highlands. The distribution of numbers of cattle per farm is highly skewed, with a median of 91 cattle and an interquartile range (IQR) of 174 (Q1–Q3: 26–200) (Fig. 1a). Over 20% of the farms reported having < 20 cattle. The number of cattle on each farm is assumed constant at the number recorded in the census.
(2) Cattle Tracing System (CTS). The Cattle Tracing System (CTS) is operated by DEFRA's British Cattle Movement Service (BCMS, 2005; Mitchell et al., 2005). In Scotland, during years 2002–2004 there were 252,496 movements among 11,464 of the cattle farms entered in the 2003 census database (the remainder are assumed not to have moved cattle to or received cattle from other farms). The data shows that there is a seasonal pattern in the numbers of cattle moved (e.g. there are more cattle movements during March–April–May and September–October–November than the rest of the year) (Fig. 1b), most movements are within 50 km (Fig. 1c) and the majority of movements involved only a few animals (Fig. 1d). Movements outside Scotland and to/from abattoirs and markets are not considered here.
(3) *E. coli* O157 prevalence data. Between February 2002 and February 2004, a cross-sectional survey was carried in Scotland funded by the Wellcome Foundation International Partnership Research Award in Veterinary Epidemiology (IPRAVE). Over the survey period, 481 farms were each visited once to obtain an estimate of the prevalence of *E. coli* O157 (Pearce et al., 2009). Farms were sampled in such a way that similar numbers were included from each of the six designated Animal Health Districts (AHD) throughout Scotland (Pearce et al., 2009) and that AHDs were sampled evenly over time. The numbers of faecal pat samples taken were chosen to ensure a mean 90% probability of detecting shedding of *E. coli* O157 if at least one shedding animal was present. Data on the CPH were not recorded in the IPRAVE survey so farms were matched to the DEFRA 2003 census data and the DEFRA CTS data for the equivalent time frame (2002–2004) using the XY coordinates. After matching, 461 of these farms were used for model fitting, among which 87 (18.9%) were positive (see Fig. 2a). The remaining 20 farms could not be matched to data in the 2003 census so they were excluded from the analysis.

## Model development

We consider a metapopulation model where individual farms are regarded as either susceptible (*S*) or infected (*I*) (i.e. *E. coli* O157 positive at least one cow). *E. coli* infection is not pathogenic in adult cattle and infections are transient (Shere et al., 1998; Robinson et al., 2004); therefore infected cattle can recover to become susceptible and *E. coli* positive farms can lose their infected status.

One route of transmission from one farm to another is the movement of infected cattle between them. Because movement data are provided for each day, it is sensible to use a time unit of one day. We assume that if a susceptible farm *i* on day *t* receives *M*_ij_(*t*) cattle from an infected farm *j* on which a fraction *x*_j_ is infected, the chance that farm *i* will become infected due to this movement event is 1 − (1 − *x*_*j*_)^*M*_*ij*_^ (c.f., Green et al., 2006). Summing over all cattle moving onto the farm from infected farms, the probability that farm *i* will become infected on day *t* is

(1) $$P_{i,\text{Move}}\left(t\right)=1-\prod_{j\in I\left(t\right)}{\left(1-x_{j}\right)}^{M_{\mathrm{ij}}\left(t\right)}\text{,}$$

where *I*(*t*) is the set of infected farms at time *t* (and also the number of infected farms at time *t*, see below). The fraction of cattle infected on farm *j*, *x*_*j*_ was randomly sampled (with replacement) from the distribution of on-farm prevalences found from IPRAVE survey data (Liu et al., 2007), and *M*_*ij*_(*t*) was obtained from DEFRA CTS data as described above.

Farms can also become infected through a variety of other routes including acquisition of infection from other host species and a contaminated environment. Let *Λ*_*i*_ represent the force of infection for farm *i* due to sources other than movements. Combining together, the overall probability that farm *i* becomes infected on day *t* is

(2) $$P_{i}\left(t\right)=1-\text{exp}\left[-\Lambda_{i}\right]\left(\prod_{j\in I\left(t\right)}{\left(1-x_{j}\right)}^{M_{\mathrm{ij}}\left(t\right)}\right)\text{.}$$

When both contributions in transmission are small, Eq. (2) can be approximated as *P*_*i*_(*t*) = *Λ*_*i*_(*t*) + ∑ _*j*_ _∈_ _*I*(*t*)_*x*_*i*_*M*_*ij*_(*t*), which suggests that the probability is proportional to the force of infection and the number of infected animals moved in. *Λ*(*t*) has units per day. The infected farms can recover to become susceptible again. For simplicity, we initially assume that this happens with a constant probability of recovery per day

(3) $$Q_{j}\left(t\right)=\gamma \text{.}$$

The expression for the force of infection *Λ*_*i*_ depends on the assumptions made about the process of transmission. As *E*. *coli* O157 is transmitted via the faecal-oral route the main transmission vehicles are thought to be contaminated feed, water and grazing. Once in the environment, *E*. *coli* O157 can remain viable for long durations (Gagliardi and Karns, 2000; Williams et al., 2005). Transmission is easier between animals kept at higher densities and hence in closer proximity (Synge et al., 2003; Smith et al., 2009). A longitudinal study of a dairy herd (Mechie et al., 1997) and previous work in Scotland (Synge et al., 2003) reported seasonal incidence of shedding. Livestock species other than cattle, and also wildlife, may be infected and transmit infection (Synge, 2000). Mechanical transmission of contaminated faeces is a likely means of farm-to-farm spread.

To represent all the possible mechanisms that are relevant to transmission, we first consider the following basic model: the probability of a farm becoming infected depends on the number of cattle present *N*_i_ and the number of infected farms at time *t*, *I*(*t*) (equivalently, the prevalence of infection across the whole population of farms) in a nonlinear way,

(4) $$\Lambda_{i}\left(t\right)=\beta N_{i}^{a}I{\left(t\right)}^{b}\text{.}$$

Here (for the case *b* = 1) *βN*_*i*_^*a*^ is the weighted probability of infection of farm *i* per infected farm per day. The dimensionless nonlinear index *b* determines the relationship between the force of infection and the fraction of farms infected (c.f., Liu et al., 1987), ranging from *b* = 0 (*Λ* independent of *I*) to *b* = 1 (*Λ* proportional to *I*) (c.f., Liu et al., 2007). The dimensionless nonlinear coefficient *a* describes heterogeneity in susceptibility as a function of the herd size, *N* (Tildesley et al., 2008). We note that the field data do not indicate any relationship between herd size and on-farm prevalence (Spearman rank correlation: *r*_s_ = −0.061, *p* = 0.574), i.e. *x*_*j*_ is not a function of *N*_*j*_.

Further, to examine the influence of other possible risk factors on the transmission of *E*. *coli* O157, the following variants to the basic model were considered:

i) *Seasonality*: the transmission coefficient varies such that *β* = *β*_c_ for December to April and *β* = *β*_w_ (> *β*_c_) for May to November.
ii) *Sheep* or *pigs*: contribution of other livestock species: the force of infection is given by*Λ*_*i*_ = *βN*_*i*_^*a*^*I*^*b*^ + *σ*, where *σ* > 0 if the other species is present and *σ* = 0 otherwise. Two sub-variants were considered: *sheep*, where *σ* refers to the presence of sheep on the farm (according to the DEFRA census data) and *pigs*, where *σ* refers to the presence of pigs.
iii) Herd size-dependent recovery rate (*N-dep-Recovery*): though the detail of the dynamical relationship between the number of cattle and the average infectious period may be complicated, here we assume simply that the recovery rate of an infected farm is dependent on the number of cattle such that *Q*_*j*_(*t*) = *γ*/*N*_*j*_^*c*^ where *c* is a constant.
iv) Imperfect farm-level detection or imperfect test sensitivity: the IPRAVE prevalence survey was designed to have a farm-level detection sensitivity of around 90% (Pearce et al., 2009) and diagnostic testing of individual samples is less than 100% sensitive (Matthews et al., 2009). To explore the effect of imperfect farm-level detection on model fitting, the model-predicted probability of a farm being observed as *E. coli* O157 positive was multiplied by 0.9 (*imperfect detection*). To explore the effect of imperfect diagnostic testing on model fitting test sensitivity was assumed (conservatively) to be just 50% (see Matthews et al., 2009) and on-farm prevalences, *x*, were accordingly transformed as (2 − *x*)*x* (*imperfect sensitivity*).
v) *No movement*: to test the importance of cattle movement for the spread of *E. coli* O157 infection, the term representing cattle movements among farms was removed from the model.
vi) *No herd size effect*: to test for any effect of herd size on susceptibility of farm we considered the special case *a* = 0.
vii) *Density-dependent* and *density-independent*: we also considered two special cases for the value of the nonlinear index *b*: *b* = 1 (corresponding to *Λ* proportional to *I*: density-dependent) and *b* = 0 (corresponding to *Λ* independent of *I*: density-independent). These correspond respectively to density-dependent and density-independent models of Liu et al. (2007).
viii) *Local spread*: we assumed that the force of infection decays exponentially with the distance (in kms) between farms at a rate *α* (i.e. so-called spatial kernel), giving
  (5) $$\Lambda_{i}=\beta N_{i}^{a}\sum_{j=1}^{I}\exp\left(-\alpha d_{\mathrm{ij}}\right)\text{,}$$
  where *d*_*ij*_ is the distance between susceptible farms *i* and infected farm *j*. To test whether there is evidence for local spread, the model was fitted with *α* set at three different values: 0.5, 1 and 2 (corresponding to increasingly localized spread). With *α* = 0, this model is equivalent to the density-dependent model above.

## Model fitting methods

The infection of *E. coli* O157 on Scottish farms is likely to be at an approximate steady state because a comparative analysis of the IPRAVE data with an early Scottish Executive Environment and Rural Affairs Department (SEERAD)-funded survey, performed between 1998 and 2000, found no statistically significant difference in farm-level prevalence of *E. coli* O157 (Pearce et al., 2009). However, the IPRAVE prevalence data are quasi-longitudinal (although individual farms were visited only once, the survey design involved a stratified sequence of visits to different regions of the country) and some fluctuation in prevalence is apparent (Fig. 2b). The time scale of these fluctuations allows us to obtain an estimate of the recovery rate.

**Fig. 3:**
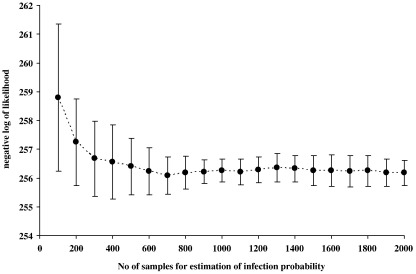
The relationship between variation in negative log-likelihood *l* and the number of samples used for model fitting. The basic model is used with *a* = *b* = 1.0 and *β* = 10^−^^8^, *γ* = 0.011 per day per farm. 12 replicates were used and the mean and standard deviation of log-likelihood calculated from these. When the number of samples is small, the values of the estimated log-likelihood fluctuate widely among different replicates. With increasing number of samples, they converge quickly. When the number of samples is greater than or equal to 1000, the values of log-likelihood are distributed across a small range, with a standard deviation of order 0.5.

We used the following method to obtain model predictions of the probability that each farm *i* is infected, *ρ̅*_*i*_, for *i* = 1 to 461. For a given model and set of parameter values, a large number of replications of simulated steady state conditions was needed to estimate the average probability *ρ̅*_*i*_ given the highly stochastic nature of the infection and recovery processes. Visual C++ was used to code the simulation programme. We randomly chose five different infection seeds, and allowed a burn-in period of 15 years to let the system reach a steady state condition (c.f. Liu et al., 2007). The model system was run for another 1000 × 3 years. *κ*–statistic tests of autocorrelation (Dohoo et al., 2003) showed that the infection status of the farm in one 3 year interval was independent of its status in the adjacent interval; thus each simulated 3 year period can be regarded as an independent sample. From these 1000 samples, the predicted probability was estimated for each individual IPRAVE farm *i* as: *ρ̅*_*i*_ = (number of times farm *i* was positive) / 1000. Here, ‘positive’ indicates that the simulation predicted that infection would be present on the day (relative to the simulated 3 year period) on which the farm was sampled in the field during 2002–2004. That 1000 samples were adequate was justified by numerical experiment. When a few samples are taken, then the average log-likelihood *l* (see below) fluctuates widely across different runs. As the number of samples increases *l* converges quickly. When the number of samples equals 1000, *l* varies over a very small range such that its standard deviation is about 0.5 units in absolute value, with no discernible reduction in variability for larger sample sizes (Fig. 3).

Given the model prediction *ρ̅*_*i*_ for each of the 461 IPRAVE survey farms, the natural logarithm of the likelihood is calculated to quantify model fit:

(6) $$l=\sum_{i=1}^{461}\log_{e}\left[{\left(1-{\bar{\rho }}_{i}\right)}^{\left(1-ri\right)}{\bar{\rho }}_{i}{}^{r_{i}}\right]\text{,}$$

where *r*_*i*_ = 1 if farm *i* is recorded in IPRAVE survey as positive, *r*_*i*_ = 0 otherwise. The downhill simplex method (Press et al., 1997) was employed to search for the maximum likelihood estimates (MLE) of the model parameters for each model. To select the most parsimonious model (balancing goodness of fit with the number of parameters fitted), the Akaike Information Criterion (AIC) (Akaike, 1973) was calculated:

(7) $$AIC_{m}=-2l\left(Data|MLE_{m}\right)+2p_{m}\text{,}$$

where *p*_*m*_ is the number of parameters of model *m* and MLE_*m*_ represents the maximum likelihood estimates of the parameters of model *m*. The most parsimonious model is the one with the lowest AIC value. To quantify the extent to which the most parsimonious model is better than competing models, evidence ratios were calculated as:

(8) $$w_{1}/w_{k}=\exp\left[\frac{1}{2}\left(AIC_{k}-AIC_{1}\right)\right]\text{,}$$

where model 1 is the estimated most parsimonious model and *k* indexes each alternative model. This value quantifies the relative likelihood that model 1 is better than model *k*, given the data set (Burnham and Anderson, 2002).

**Fig. 4:**
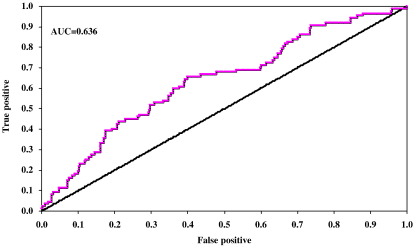
The ROC curve for the comparison between IPRAVE samples and prediction of the basic stochastic model with best fit parameters (see Table 1). The X- and Y- axes are false positive rate and the true positive rate, respectively.

To evaluate each model's accuracy in predicting the observed infection status of each individual IPRAVE farm, an empirical estimate of the odds ratio for each independent sample was calculated by:

(9) $$OR=\frac{F_{++}\times F_{--}}{F_{+-}\times F_{-+}}\text{,}$$

where *F*_++_ is the number of farms that are positive for both model prediction and observed data, and the meanings of *F*_--_, *F*_+-_ and *F*_-+_ follow accordingly. An odds ratio greater than 1 indicates that the model is more likely than not to predict the correct infection status of a farm. The larger the odds ratio, the stronger the predictive power of the model. We also calculate the area under the Receiver Operating Characteristic (ROC) curve, which provides a measure of a model's ability to discriminate between farms that are positive and those that are negative (Dohoo et al., 2003). For this (non-parametric) calculation, the farms are arranged in the descending order of the predicted probability *ρ̅*_*i*_, which acts as the cut-off point for distinguishing between positive and negative farms. In our system, 461 farms were sampled, of which 87 positive. For a given value of cut-off point, say *ρ*_CT_, the false positive rate (FPR) is calculated as *N*_FP_/374 where *N*_FP_ is the number of farms that are actually negative and have predicted probability *ρ̅*_*i*_ ≥ *ρ*_CT_, the true positive rate (TPR) as *N*_TP_/87 where *N*_TP_ is the number of farms that are actually positive and have predicted probability *ρ̅*_*i*_ ≥ *ρ*_CT_. We obtain the Receiver Operating Characteristic (ROC) curve by plotting TPR versus FPR over all possible *ρ*_CT_ values from which the area under the ROC curve (AUC) can be derived (Fig. 4).

## Analysis of risk factors

Data for 461 of the 481 farms sampled in the IPRAVE study were extracted from the DEFRA CTS database (time of movement events, number of animals moved, coordinates of destination farm) during year 2002–2004 and the DEFRA 2003 census database (the X–Y coordinates of the farm-house, the area of the farm, and the numbers of cattle, sheep and pigs) as described above. Additional variables were created to describe farm clustering (number/presence of farms within 1, 3 and 5 km), characteristics of surrounding farms (size, type, distance, number of cattle), recent movement (movement within 1, 2, 3, 4, 8, and 30 weeks before sampling), amount of movement (number of movement ‘events’ onto farms, number of cattle moved, number of different farms supplying cattle). Movement events were further characterised by distance moved (< 10 km, > 10 km), number of animals moved per ‘event’ and the season of movement. Risk factors for the presence of *E. coli* O157 on a farm were analysed using generalised linear models (GLM) and Generalised Linear Mixed Models (GLMM) procedures in SAS (SAS Institute Inc., Cary, NC) with a binomial error distribution and a logit link function. GLM analyses were carried out on a univariate basis initially. A hierarchical forward selection and backward elimination approach was used. The change in the deviance of the model was monitored as an indicator of improved fit. Variables were added and removed based on significant improvement in the mean deviance after changes to the model. Two-way interactions were also tested in this manner. The final model was carried forward for GLMM analysis incorporating a random variable called ‘farm cluster’ which records the temporal/spatial nature of the sampling carried out during the IPRAVE survey.

In order to compare the results of the statistical analysis to the results generated from the stochastic modelling an analogous empirical odds ratio estimate was developed. Values for the empirical estimate of the odds ratio were derived using the parameter estimates from the GLMM model. These estimates were used in the statistical model to simulate binary response random variables for each farm (absence/presence of *E. coli* O157 on-farm). The predicted presences/absences were then related to the observed presences/absences, and aggregated over all farms, from which summaries an odds ratio was calculated as above (Eq. (9)). This process was repeated to produce a distribution of odds ratios which could then be summarised.

During a survey from March 1998 to May 2000, funded by The Scottish Executive Environment and Rural Affair Department (SEERAD), 952 farms were surveyed. It was found that *E. coli* O157 is present on 21.7% (19.2–24.5%) of the farms (Gunn et al., 2007). Among 461 IPRAVE farms of interest here, 395 farms were also in the SEERAD study. As a test of the predictive power of the most parsimonious model (see Results), the expected degree of association (using Cohen's kappa statistic, *κ* — Cohen, 1960) between different sets of simulation results for those 395 farms was calculated and compared to the observed degree of association.

## Results

Table 1 shows the comparison among different model variants. According to the AIC value the most parsimonious model is the basic model, i.e. that described by Eqs. (2)–(4). We therefore compare all other models with the basic model (noting that there is some error in likelihood estimates – see Fig. 3 – and therefore in the AIC values).

i) The basic model with nonlinear index *b* fixed at 0 is very close to the most parsimonious model, and the likelihood ratio test (LRT = 2.4 with standard error 0.40) suggests that they are not significantly different at a 5% significance level. In contrast, the basic model with nonlinear index *b* fixed at 1 is significantly different (LRT = 5.6 with standard error 0.40) and this model can be rejected at the a 5% significant level.
ii) The modifications to the basic model that allow for the imperfect detection of infection at the farm level (imperfect detection) or imperfect diagnostic test sensitivity at the sample level (imperfect sensitivity) do not improve model fit and although both result in some modification of model parameter estimates these remain within the ranges estimated from other plausible models. Hence we do not consider these modifications further.
iii) The models with an extra parameter for the presence or absence of pigs or sheep both have higher AIC values and neither is preferred to the basic model (evidence ratios > 2 and > 4 respectively). For the model incorporating sheep no improvement was found over the basic model.
iv) The model with seasonal transmission also incorporates one extra parameter but there was no improvement in fit over the basic model and so this model has a higher AIC value and is not preferred (evidence ratio = 3).
v) The model with a recovery rate dependent on farm size has a much higher AIC value, an evidence ratio > 20, and can be rejected.
vi) The model without an effect of herd size on susceptibility (*a* = 0) has a much higher AIC value, an evidence ratio > 20, and can be rejected.
vii) The model without movement-related spread of infection has a much higher AIC value, an evidence ratio > 60, and can be rejected.
viii) The models including a spatial kernel do not improve model fit: with larger values of *α* (i.e. more localized spread of infection), the likelihood dramatically decreases and the AIC value substantially increases. These models can be rejected.

The most parsimonious model generates a predicted prevalence of infection on the IPRAVE study farms close to the observed prevalence of 18.9% and well within the exact binomial 95% confidence intervals (15.5 to 22.7%). However, the mean odds ratio (which gives a measure of the ability to distinguish between positive and negative farms) is low (1.38, with a standard error of 0.39) and the area under the ROC curve (AUC) is only 0.636 (Fig. 4) (below the 0.7 threshold for ‘acceptable’ discrimination specified by Hosmer and Lemeshow, 2000). Several of the alternative models perform marginally better using one or more of these indicators but in general the models perform poorly at discriminating between positive and negative farms.

The models can also be compared in terms of the parameter estimates obtained. Estimated values of *β* are highly dependent on model structure and so vary widely between models. Estimated values of *γ* (with the exception of the model relating recovery rate to farm size) are not directly influenced by model structure and show much less variation. For the best five models estimates of *γ* range from 0.0232 to 0.0354. Similarly, estimates of *a* range from 0.249 to 0.476 and of *b* (unless fixed) from 0.176 to 0.230. Overall, where comparable, parameter estimates are reasonably robust across the set of best models.

**Fig. 5:**
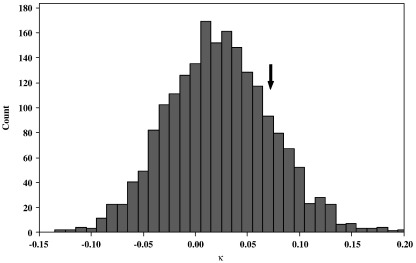
The distribution of Cohen's *κ* values between different sampling periods as predicted by the most parsimonious simulation model. The modal value is 0.01. The observed value of *κ* for the SEERAD and IPRAVE surveys is 0.077 (indicated by the arrow).

The results of comparing the simulation output for different time periods show that the model predictions for the periods 1998 to 2000 and 2002 to 2004 are only weakly associated: the modal value of *κ* is 0.010 (Fig. 5), i.e. slightly larger than random. Comparison of the SEERAD and IPRAVE survey data gives a similarly low *κ* value. Among the 395 farms, 22 farms were positive and 253 were negative in both surveys, while 56 farms were positive in the SEERAD survey and negative in the IPRAVE survey, and 64 farms were negative in the SEERAD survey and positive in the IPRAVE survey giving *κ* = 0.077 (95% confidence interval −0.028 to 0.182, *p* = 0.128).

The results of the statistical analysis of the GLMM model are shown in Table 2. The results suggest that farms that were positive for *E. coli* O157 tended to have a larger number of cattle as well as recent movements of cattle (within 4 weeks of sampling) onto the farm. The mean overall odds ratio for this model was 1.297, with a standard deviation of 0.379. None of the variables related to the clustering of farms or the presence of sheep were statistically significant in the multi-variable models. These results are very similar to the results obtained by the basic model in the simulation models above.

## Discussion

To investigate factors that affect the transmission dynamics of *E. coli* O157 infection on Scottish cattle farms, we developed a set of stochastic models and employed AIC to select the most parsimonious model. By comparing the fit of different model variants, we have shown that the most parsimonious model to describe the dynamics of the spread of *E. coli* O157 infection between farms is the one that is given by Eqs. (2)–(4). This model simply says that the transmission between farms is by two different routes: i) movement of infected cattle; and ii) all other routes, including acquisition of infection from a contaminated environment or other reservoir. The recovery rate is a constant, but the farm susceptibility increases with herd size (c.f. Tildesley et al. (2008) who found a similar result for farm susceptibility to foot-and-mouth disease). The force of infection (ignoring transmission via movements) is not a linear function of the number of infected farms (contrasting with the standard mass action assumption). The nonlinearity (with coefficient *b* considerably below 1) indicates saturation, that is, the risk that a susceptible farm becomes infected increases more rapidly with each additional infected farm when there are few infected farms than it does when there are many (c.f. Liu et al., 1987). Indeed, we cannot formally exclude the possibility that the force of infection is independent of the number of infected farms, i.e. *b* = 0. This result has a significant influence on the anticipated effectiveness of intervention measures, explored in detail elsewhere.

More complex models incorporating other risk factors do not significantly improve the fit. For example, because sheep and pigs can also carry *E. coli* O157, the presence or absence of these other livestock species could give rise to different probabilities of infection. Similarly, the prevalence of infection varies seasonally, so season could also affect transmission rate. Inclusion of these risk factors marginally improves the model fit but increases the AIC value, so the models are rejected as not parsimonious. Also, it is plausible to argue that proximity to an infected farm makes a farm more likely to become infected. However, our model fitting exercise provided no support for localized spread. Further, it is possible that a large farm will remain infected for longer and has a smaller recovery rate but this does not improve model fit, and thus was rejected.

All of these results are consistent with our risk factor analysis. Generalised Linear Mixed Models also identify herd size and cattle movements as risk factors, but the other risk factors considered here (sheep, seasonality, clustering of farms) do not have statistically significant effects. Correspondence between the results from empirical GLMMs and those from the fitting of dynamical models has not, as far as we are aware, previously been examined for any infectious diseases system. The good agreement found here is encouraging since both approaches are commonly used to inform the design of disease control programmes, which implies that the targeting of interventions could be based on either (this point will be considered in more detail in subsequent work).

Model selection, not parameter estimation, was the major objective of this analysis. The maximum likelihood method used here does not readily yield confidence intervals on parameter estimates; these are being obtained using Markov chain Monte Carlo methods and will be reported separately. However, we note that the estimates obtained appear reasonably robust across the best fitting set of models, which includes two models allowing for the slight under-detection of infection allowed for in the field survey design and for the imperfect diagnostic test sensitivity. The estimated rate of loss of infection gives an average duration of infection on a farm of approximately one month; this is highly consistent with data from smaller scale longitudinal studies (Synge et al., 2003).

Although these analyses do suggest farm size and cattle movements as significant risk factors the risk profile across the population of farms is relatively flat. This is indicated by the low odds ratios (Tables 1 and 2) and the small area under the ROC curve (Fig. 4). Reflecting these results, model simulations indicate that, while less than 20% farms are *E. coli* positive on any given visit, over 80% can be expected to be infected at some stage during a calendar year, reflecting both the risk profile and the typically short duration of infection on a farm (Robinson et al., 2004). This prediction is testable and clearly has important consequences for the design of future control programmes.

## Funding

This work was supported by the Wellcome Trust (project grant to MEJW) and the DEFRA/SFC VTRI programme.

## Conflict of interest

The authors do not have commercial or other associations that might pose a conflict of interest.

## Author contributions

X-SZ performed all the process modelling and wrote the paper. MECT performed the statistical modelling and prepared the manuscript for submission to the journal. IJM designed the analysis to generate comparable AUC estimates from the empirical modelling and helped to interpret the data. MEJW and NJS supervised the study and helped to interpret the data. All authors read and approved the final manuscript.

## Figures and Tables

**Table 1:** Comparison between models. The model variants are listed in the ascending order of their AIC values. *l* is the natural log of likelihood calculated using Eq. (6), *I*prev is the prevalence of infection on 461 IPRAVE farms, AIC is the Akaike Information Criterion (Eq. (7)) and *w*_1_*/w*_*k*_ is the evidence ratio (Eq. (8)). *OR* is the odds ratio of the model prediction (Eq. (9)).

Model variants	*β*^#^	*γ*	*a*	*b*	Additional parameter	*−l*^§^	*I*prev^†^	AIC^§^	*w*_1_*/w*_*k*_	*OR*
Basic model	3.38e-4	3.50e-2	0.270	0.230	–	213.0	19.4	434.0	1.00	1.38
Density-independent	2.40e-3	3.54e-2	0.249	0.0	–	214.2	19.8	434.4	1.25	1.26
Imperfect detection	1.27e-4	2.32e-2	0.437	0.188	–	213.4	19.7	434.8	1.48	1.48
Imperfect sensitivity	2.91e-4	3.35e-2	0.317	0.212	–	213.7	19.4	435.4	2.01	1.32
Pig	1.26e-4	2.82e–2	0.476	0.176	*σ* = 1.15e-2	212.9	18.2	435.8	2.48	1.51
Seasonality	2.72e-4	2.84e-2	0.292	0.196	*β*_w_ = 3.22e-4	213.1	19.2	436.2	3.00	1.42
Sheep	1.95e-4	2.94e-2	0.396	0.189	*σ* = 8.78e-4	213.6	20.1	437.2	4.95	1.43
Density-dependent	1.48e-6	5.14e-2	0.248	1.0	–	215.9	17.2	437.7	6.55	1.23
No herd size effect	3.15e-4	4.38e-2	0.0	0.437	–	217.0	19.5	440.0	20.7	1.25
*N*-dep-recovery	2.28e-4	0.112	0.187	0.329	*c* = 0.242	215.1	18.8	440.3	23.1	1.33
No movement	1.73e-4	3.16e-2	0.379	0.246	–	217.2	18.2	442.4	68.0	1.20
Local spread	3.32e-5	1.94e-2	0.87	–	α = 0.5	262.6	22.6	531.2	1.32e+21	1.71
	9.25e-5	1.89e-2	0.901	–	α = 1.0	272.8	19.6	551.5	3.34e+25	1.65
	2.36e-3	1.20e-2	0.459	–	α = 2.0	277.1	23.8	560.2	2.61e+27	1.42

^#^For model variant Seasonality, the value of *β* listed in this column is *β*_c_ for December to April. ^§^The standard errors in estimates of −*l* and AIC are approximately 0.15 and 0.30 respectively (Fig. 3). ^†^The prevalence of infection for the whole system is about 2–3% lower than *I*prev.

**Table 2:** Results of the GLMM statistical models of risk factors for the presence of *E. coli* O157 on the 461 IPRAVE farms. Overall OR gives empirical estimate of odds ratio for the entire model.

Predictors	Estimate	SE	*p*	Overall OR^a^
Movement within 4 weeks	0.664	0.288	0.0214	
Number of cattle^b^	0.785	0.322	0.0151	
				1.297 (0.379)

a Mean (SD).

b Log_10_ transformed.
